# Preventing Violence against Healthcare Workers in Hospital Settings: A Systematic Review of Nonpharmacological Interventions

**DOI:** 10.1155/2023/3239640

**Published:** 2023-12-06

**Authors:** Natasha Mundey, Victoria Terry, Jeff Gow, Jed Duff, Nicholas Ralph

**Affiliations:** ^1^School of Nursing and Midwifery, University of Southern Queensland, Toowoomba, Australia; ^2^Centre for Health Research, University of Southern Queensland, Ipswich, Australia; ^3^School of Business, University of Southern Queensland, Toowoomba, Australia; ^4^School of Accounting, Economics and Finance, University of Kwazulu-Natal, Durban, South Africa; ^5^School of Nursing, Queensland University of Technology, Kelvin Grove, Australia; ^6^Faculty of Health, University of the Sunshine Coast, Moreton Bay, Australia

## Abstract

**Background:**

Up to 92% of health workers experience some form of patient-perpetrated violence. The highest risk environments include emergency departments, acute care settings, and mental health units. Given such elevated rates of violence, current interventions have questionable efficacy or implementation challenges.

**Design:**

We conducted a systematic review conforming to PRISMA reporting requirements. We searched PubMed, CINAHL, PsycINFO, Scopus, and the Cochrane Library. Studies reporting interventions to prevent patient-initiated violence against healthcare workers in hospitals were included, and findings were synthesised.

**Results:**

Based on meeting eligibility criteria, twelve studies were included in the review. Most interventions reported an effect with eleven of the twelve studies describing changes in the incidence of violence postintervention. Most studies were evaluations of education and training programs (*n* = 7), followed by action plans (*n* = 2), and a reporting instrument, risk assessment tool, and legislation (*n* = 1).

**Conclusions:**

Insights into effective strategies to prevent hospital patient and visitor-initiated violence are necessary to develop guidelines for better aggression/violence deterrence. Violence prevention requires strong, evidence-based, and clinically applicable interventions that promote the safety and satisfaction of all healthcare workers. *Relevance to Clinical Practice*. Formulating effective and appropriate strategies that aid in early recognition, prevention, and management of aggression/violence will benefit all health workers. Patient and staff satisfaction will rise; healthcare workers will regain a sense of preparedness, and higher levels of safety will be achieved. Without these effective interventions being established, the magnitude of adverse outcomes from patient-perpetrated violence will continue in healthcare.

## 1. Background

The World Health Organisation (WHO) defines violence as “the intentional use of physical force or power, threatened or actual, against oneself, another person, or against a group or community, that either results in or has a high likelihood of resulting in injury, death, psychological harm, maldevelopment or deprivation” [1]. WHO reports that violence in healthcare settings represents about a quarter of violence in all workplaces and that violence against healthcare workers is a worldwide problem [2]. More than 50% of those responding to this seven-country study reported at least one incident of physical or nonphysical violence in the preceding year. A more recent systemic review indicated that the 12-month prevalence of any form of physical or nonphysical violence against healthcare workers was 61% globally [3].

Healthcare workers experience patient- or visitor-perpetrated violence at some stage in their careers [4], resulting in minor to major physical injury, death, psychological trauma, mental health concerns, and/or emotional burdens such as anger, sadness, fear, and disgust [5–7]. Likewise, the healthcare environment poses a risk of exposure to violence like frontline police personnel, with the healthcare sector accounting for more than half of all violent incidents reported in workplaces [8, 9]. Workplace violence in healthcare predominantly occurs in “high risk” areas including emergency departments, psychiatric units, intensive care units, obstetrics and gynaecology, and acute medical/surgical units [10], yet the epidemiology of violent patient behaviours varies greatly. Contributing factors associated with heightened aggression include the haemodynamic instability of patients, their impaired neurological status, employee workloads, lack of experience, and burnout and environmental demands in hospital contexts [4, 10–13].

With such a range of patient, professional and organisational factors arguably inherent or inevitable in healthcare contexts, it is important to better understand and prevent violence by reporting and responding to it. Nonetheless, actual rates of violence may be higher than evidence suggests, as many studies indicate an underreporting phenomenon due to numerous factors including confusion over what constitutes violence [8], time constraints preventing disclosure [8, 14, 15], fear of repercussions [5], and the belief that violence is merely “part of the job” [16].

Recently, serious incidences of violence in healthcare have been covered broadly in the media, and in some instances, fatalities have been reported. For example, an Australian surgeon was fatally punched by a patient [17], an American mental health patient was charged with the manslaughter of a nurse [18], in China, a doctor was stabbed to death by a patient's family member [19], and in South Africa, an anaesthetist was fatally shot in an apparent revenge attack following the death of a child during surgery [20]. Likewise, serious assaults are also common. An Australian nurse was knocked unconscious and required cardiopulmonary resuscitation following a patient attack [21]; in the United States, a nurse had their stethoscope wrapped around their neck and was forced to defend herself [22], and England's chief nurse called for an end to physical and verbal assaults on nurses as they worked through the COVID-19 pandemic [23].

Physical injuries such as bruising, cuts, and abrasions are the most reported injuries from violence in hospitals [5], although the psychological burden on healthcare workers following an assault includes fear, anger, anxiety, guilt, shame, self-blame, reduced job satisfaction, increased desire to change employment, and a reduction in health-related quality of life [6, 7]. Organisationally, violence is also associated with an increased risk of absenteeism, lowered morale, and decreased productivity amongst healthcare workers [24–26]. The economic cost of violence in healthcare is significant with treatment, leave expenses, staff burnout, compensation, increased turnover, and security, litigation, and property repair expenses burdening the sector [8, 9, 11, 27].

Despite its impacts, few studies have explored the efficacy of interventions to prevent violence. Whilst some studies have explored the role of pharmacological strategies like sedating or restraining violent patients [5, 8, 15, 28], drug-based therapeutic solutions [5, 15, 28] are not a leading solution to workplace violence in healthcare and can lead to serious consequences for patients [5, 15, 29]. Currently, no systematic reviews have been conducted to measure the effectiveness of nonpharmacological interventions to prevent patient-perpetrated violence against healthcare professionals within hospital settings. Key objectives of this review include identifying interventions effective to prevent violence and evaluating the extent of evidence to support specific preventive approaches to violence. We considered any nonpharmacological intervention as those which included but were not limited to education and training, human resourcing interventions such as staffing, security interventions, environmental modification, policies and procedures, and behaviour contracts.

### 1.1. Aim

The aim of this systematic review is to analyse nonpharmacological interventions for the prevention and management of violence towards healthcare workers by patients and visitors.

### 1.2. Methods

#### 1.2.1. Design

Recommendations from the Preferred Reporting Items for Systematic Review and Meta-Analysis Protocols (PRISMA-P) 2015 statements have been used to develop the methods for undertaking this systematic review [30]. The systematic review protocol has been registered in the International Prospective Register of Systematic Reviews (PROSPERO) CRD42017065700.

#### 1.2.2. Inclusion and Exclusion Criteria

Any interventional study that focused on preventing violence in a population of adult hospital patients or visitors of 18 years of age and older was eligible for inclusion (see Table 1). Interventions included were those designed with the stated objective of preventing or reducing the incidence of violence/aggression perpetrated by patients or visitors in the hospital. Studies were not restricted based on design; however, studies were required to have a comparison group of some kind. Outcomes were focused on the effectiveness of the intervention on deescalation, detection of the risk of violence, frequency of violence, injury, and the use of restraint/seclusion. This review only focused on reported acts of violence or aggression committed by patients or visitors within the hospital setting.

#### 1.2.3. Search Strategy

**Figure 1:**
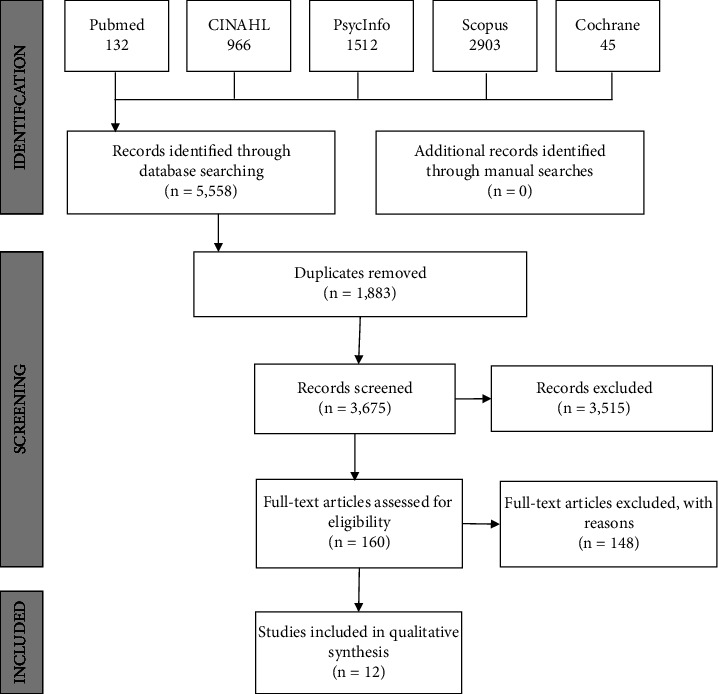
PRISMA flowchart.

Scopus, PubMed, Cumulative Index to Nursing and Allied Health Literature (CINAHL), PsycINFO, and the Cochrane Library were searched. The reference list of included articles was also read to search for additional potential studies for review. The search strategy is described in Table 2. All imported data from the search strategies were stored in EndNote with duplicates removed. Initially, 5,558 citations were retrieved, and of these, 1,883 were removed via EndNote as duplications. The remaining 3,675 were then screened for eligibility independently by two authors (NM, NR). A total of 160 full-text articles were retrieved for detailed scrutiny to further assess eligibility. Twelve articles met all inclusion criteria and were selected for review.  Table 3 provides a summary of those full-text studies reviewed for inclusion and those excluded, and Figure 1 provides an outline of the screening process.

#### 1.2.4. Data Extraction

Included studies were categorized into groups based on key interventions. Additional information extracted included the type and size of the study, participant groups, outcomes, and the risk of bias score calculated. Data were independently extracted and checked for concordance between reviewers (NR, NM) using a data extraction tool with a minimum of 95% concordance for extraction accuracy to ensure the completion of data.

#### 1.2.5. Quality Assessment

In systematic reviews, especially those involving health services research, the appraisal of methodological quality and quality of reporting is crucial. Traditionally, separate tools have been used for quantitative and qualitative research, owing to the distinct nature of these methodologies which poses challenges in the cohesion of evidence synthesis. The Quality Assessment for Diverse Studies (QuADS) tool addresses this gap by providing a standardized, empirically grounded tool suitable for a variety of study designs, including mixed-methods research [36]. The QuADS tool was used as it demonstrates strong reliability and ease of use for application to multi- or mixed-methods health services research reviews [36]. The quality assessment was performed independently by two reviewers (NM and NR) who followed the six-step procedure outlined in the QuADS criteria with a kappa value of 0.91 indicating excellent agreement with criterion-based quality assessment performed for each paper against the 13 different QuADS criteria independently (see Table 4). Any discrepancies were resolved through collaborative discussion.

#### 1.2.6. Data Synthesis

There was marked methodological heterogeneity in the studies, and as a result, narrative synthesis was chosen as the most appropriate method to analyse and explain findings. Narrative synthesis involved a modified version of the framework developed to investigate findings and to explore relationships within and between the data [37]. This process involved tabulating study data under headings that included participants, intervention, comparisons, outcomes, and themes. Tabulation allowed for consideration of themes related to key interventions, the development of textual descriptions, and the exploration of relationships within and between studies for an overall assessment of the strength of evidence [37].

## 2. Results

Of the twelve articles included in this review, eleven reported a change in the incidence of violence postintervention. Various design methods were utilised including before and after study design [8, 27]; randomised controlled trial [15]; quasiexperimental studies [29, 31, 34]; literature review and intervention establishment [11]; qualitative quality improvement observational study [10]; investigative study [32]; a risk assessment checklist and preventative protocol [34]; cross-sectional surveys [33, 35], and chart review and assessment [9].

The 12 interventions included education and training programs (7): [8, 10, 29, 31, 34, 35, 38]; action plans (2): [11, 15]; detection instrument (1): [27]; risk reporting tool (1): [9], and legislation (1): [33]. Specific settings such as emergency departments [10, 27, 33–35] and obstetrics and gynaecology units [29] were identified along with those studies which targeted multiple units and the entirety of the hospital [15].

Study populations included healthcare employees: nurses [8, 9, 31, 34]; doctors and medical students [10, 35]; human resource representatives, security personnel and administration staff [11, 35]; general/unspecified healthcare workers [9, 15, 29, 33, 38]; and patients [27]. Studies were conducted in Australia (1), France (1), Iran (3), Pakistan (1), and the United States (6).

Regardless of the variations between intervention types, population groups, and design methods, each study reported on the incidence of physical and/or verbal violence either as a combined or singular phenomenon. Five outcomes of interest were reported across the twelve studies for inclusion as follows: “Frequency of Violence,” “Capability to De-escalate,” “Screening for Risk of Violence,” “Injury,” and “Need for Restraint/Seclusion.”

### 2.1. Interventions for Decreasing the Frequency of Violence

Overall, ten of the twelve studies noted a decrease in the frequency of violence postintervention whilst two reported no statistically significant difference [11, 29]. Of the ten, one also noted a decrease in the incidence of patient-to-patient violence and the use of seclusion/restraints but no change in the rate of patient-to-staff violence [32]. Reductions in the incidence of violence ranged from 23% to 91.6% [10, 27], respectively. Characteristics of interventions to diminish violence included education programs, action plan development for violence prevention, deescalation and nonviolent crisis intervention training, violence reporting instruments, system efficacy examination, virtual training and questionnaires, risk assessment checklist, and a preventative protocol.

### 2.2. Interventions for Improving Staff Capability to Deescalate

Three studies reported the use of deescalation techniques to reduce violence, actual, or potential [8, 11, 29]. Current deescalation techniques focus on the adaptation of healthcare providers' behaviours to better respond to, prevent, and reduce violence including the use of self-defence training, verbal deescalation strategies, training programs, and the implementation of the Confidence in Coping with Patient Aggression Instrument (CCPAI). Characteristics of interventions utilised in these studies included two training programs and one education program. Of these studies, two reported results to be statistically nonsignificant, mixed, and inconclusive [11, 29]. The final study noted the use of verbal deescalation strategies significantly increased (*p* = 0.011), resulting in diminished frequency and recurrence of violence by 45% [8].

### 2.3. Interventions for Screening for Risk of Violence

Three studies utilised screening assessment tools to aid in the identification and perception of potentially violent patients [8, 9, 34]. One of these studies reported an overall reasonable effectiveness in identifying those most at risk of committing violence with moderate sensitivity (71%) and high specificity (94%) [9]. The second study noted decreased frequency and recurrence of violent incidents [8], whilst the third noted a reduction in the perceived risk of violence from 46 to 35% [34]. Characteristics of interventions screening for the risk of violence included the Alert Assessment Form protocol, and the use of specific characteristic criteria to recognise those patients most at risk [8, 9, 34].

### 2.4. Interventions to Reduce Violence-Related Injury

Two studies reported a decrease in the frequency of injury as a direct result of violence. One noted the rate of incidents decreased by 91.6% with staff visits to the medical centre reducing by 42.2% [27]. The second study had minimal impact on the occurrence of self-defence-related injuries in participants with pre- and postintervention rates of 37.5% and 35.7% [35]. These study interventions were characterised by the creation of an instrument allowing employees to better report threats and acts of violence which directly impeded on patient care.

### 2.5. Interventions to Reduce the Need for Restraint/Seclusion

Only one study highlighted a reduction in the use of seclusions and restraints [32]. The characteristics of the intervention diminished the need for restraining and secluding patients by examining the efficacy of the Mandt System.

## 3. Discussion

Importantly, following a systematic review of interventions for preventing violence in hospitalised patients, we identified ten of the twelve included studies reported a decrease in the frequency of violence following the introduction of an intervention. However, with the current incidence of violence of concern in the literature, we note this review identified only 12 studies of predominantly low quality. Nonetheless, the studies reported on a diverse range of effective interventions for improving health professionals' capabilities to deescalate violence in hospitals; screening for risk of violence; reducing the frequency of violence; preventing or reducing injuries from violence; and lowering the need for restraint/seclusion.

Deescalation techniques predominantly adopt a “prevention is better than a cure” approach to violence management. Education-based training strategies are commonly utilised and readily adopted into practice by clinical staff [38]. These interventions report an enhanced knowledge of self-defence, break-away techniques, and confidence in aggression management tactics [39–41]. Regardless of their successes, these studies were of low quality with reporting methods being inconsistent. In noting this, it only reiterates that despite the best efforts to teach, train, and educate healthcare workers, current deescalation techniques are inadequately established.

Screening for potentially violent patients aids in the facilitation of early intervention, prevention, and management [13]. Questionnaires, observational checklists, and risk identification screening tools are frequently used to assist in the recognition of high-risk patients [42–44]. Such interventions have commendable success rates; however, they continue to fail when used as a stand-alone measure, and a general lack of consensus remains regarding follow-up instructions [42]. Therefore, it can be concluded that without the formulation of supplemental instructions, screening tools alone will prove insufficient.

Multiple interventions have been formulated to assist in the decrease of patient-perpetrated aggression. Much of the literature highlights that targeted, clinically applicable, and effective solutions are needed to assist in the identification, prevention, and reduction of workplace violence [45, 46]. Despite this, current methods remain substandard irrespective of their optimistic impact [47]. Until such a time that workplace-associated violence is adequately addressed, it can be rationalised that patients will continue to burden the healthcare sector with acts of aggression.

When evaluating the net benefit of establishing interventions for workplace violence management and prevention against leaving practice unchanged, the answer is obvious. The financial strain it places on the hospital sector is immense [48]. Likewise, psychological, physical, emotional, and social repercussions to staff leave them under significant pressure and unable to fulfil their role-required responsibilities [49, 50]. Immediate action through the establishment of quality interventions is fundamental to bringing positive change to the healthcare sector. By doing so, staff injury rates have the potential to decrease, proving beneficial to the workforce.

The use of seclusion and restraint of patients, physical or chemical, remains highly controversial and problematic [51]. Although it can be argued that these measures can improve patient and staff safety [52], many have serious negative repercussions and create ethical challenges in the delivery of care. Increased stress and agitation, loss of independence, restricted movement, haemodynamic instabilities, and deprivation of dignity have all been reported as adverse outcomes following restraints and seclusion [53].

Many studies have proposed the use of education-based interventions to reduce the need for such “invasive” measures. Seclusion and restraint (chemical and/or physical) of patients remain, both legally and ethically [51]. Whilst the direct and indirect use of these measures can be somewhat justified by maintaining safety [52], they can pose serious negative consequences. Increased agitation and stress, loss of independence and ability to move, the development and exacerbation of pressure area injuries, haemodynamic instability, and deprivation of dignity are all reported adverse outcomes from the use of restraint and seclusion [53]. It has, therefore, been proposed that through education and awareness, the use of such measures can be reduced whilst maintaining a safe environment for all [53]. In doing so, the perceived deprivation of basic human rights is lowered, and it becomes less distressing for patients, their families, and staff and facilitates a more positive environment for all.

Across all studies, we found that studies were of low quality and that no consistent outcome measures and reporting or definitions of violence were used. Studies of various designs and interventions suggest that further work is needed in designing high-quality studies to robustly evaluate interventions that aim to prevent violence in hospitals. Further research is, therefore, needed to develop high-quality interventions using recognised methodologies and test-developed interventions using rigorous designs.

This review is the first of its kind in collating and synthesising the available evidence on the important topic of violence prevention. Conclusively, the deterrence of patient-perpetrated violence benefits the entirety of the healthcare system. Unnecessary expenses are reduced, workforce retention improves, the safety of staff, patients, and visitors is maintained, and the overall functionality of the hospital is enhanced. Despite some promising interventions presented in the literature, the quality of the reporting of such remains inconsistent as per our quality assessment. Moreover, the literature is scant in the context of frequent violence in healthcare settings. Multiple evidence gaps were noted which require urgent investigation to ensure the formulation of evidence-based policies and the implementation of effective interventions into practice.

## 4. Implications for Nursing Management

This systematic review highlights the need for nurse managers to prioritize the development and implementation of evidence-based strategies to mitigate violence in healthcare settings. Nursing leaders are encouraged to actively engage in the development and enforcement of robust violence prevention protocols, focusing on a multifaceted approach that includes education-based interventions, effective deescalation techniques, and rigorous screening tools for potential violence. Emphasis should be placed on continuous training and support for clinical staff, fostering a culture of safety and preparedness against patient-perpetrated aggression. Furthermore, nursing management must advocate for high-quality research to refine existing interventions and explore novel strategies. By doing so, not only can the incidence of violence be reduced but also the physical and psychological well-being of healthcare professionals can be safeguarded, ultimately enhancing patient care and overall hospital functionality. The role of nursing management is pivotal in bridging the gap between current practices and the ideal state of violence-free healthcare environments, ensuring the safety and dignity of both staff and patients alike.

## Figures and Tables

**Table 1:** Inclusion and exclusion criteria.

*Inclusion criteria*

Interventional studies on preventing violence against health professionals in hospital patients
Adult human patients (≥18 years)
Published peer-reviewed articles

*Exclusion criteria*
Publication type
Narrative reviews
Editorials
Government reports
Books or book chapters
Commentaries
Consensus statements
Unpublished manuscripts
Clinical guidelines
Lectures and presentations
Study population
Animals
Children
Pregnant women
Forensic patients
Forensic psychiatric patients
Diagnosed psychiatric illness/patients
Study setting
Nonhospital settings

**Table 2:** Preliminary search strategy for systematic review.

Database	Search strategy
Scopus 2903 retrieved	(KEY (violence) OR KEY (aggression)) AND (TITLE-ABS (intervention) OR TITLE-ABS (managing) OR TITLE-ABS (prevent^*∗*^) OR TITLE-ABS (management) OR TITLE-ABS (de AND escalation) OR TITLE-ABS (reducing)) AND (TITLE-ABS (hospital) OR TITLE-ABS (ward) OR TITLE-ABS (unit)) AND (LIMIT-TO (DOCTYPE, “ar”) OR LIMIT-TO (DOCTYPE, “re”) OR LIMIT-TO (DOCTYPE, “ip”)) AND (LIMIT-TO (LANGUAGE, “English”)) AND (LIMIT-TO (DOCTYPE, “ar”) OR LIMIT-TO (DOCTYPE, “re”) OR LIMIT-TO (DOCTYPE, “ip”)) AND (LIMIT-TO (EXACTKEYWORD, “Human”)) AND (EXCLUDE (SUBJAREA, “BIOC”) OR EXCLUDE (SUBJAREA, “DENT”))

PubMed 132 retrieved	((((“violence”[MeSH Major Topic]) AND “aggression” [MeSH Major Topic])) AND (((((((((“treating” [Title/Abstract]) OR “intervention” [Title/Abstract]) OR “interventions” [Title/Abstract]) OR “managing” [Title/Abstract]) OR “prevention” [Title/Abstract]) OR “preventing” [Title/Abstract]) OR “management” [Title/Abstract]) OR “de-escalation” [Title/Abstract]) OR “reducing” [Title/Abstract])) AND (((((“hospital”[Title/Abstract]) OR “clinic” [Title/Abstract]) OR “ward” [Title/Abstract]) OR “unit”[Title/Abstract]) OR “department”[Title/Abstract])

CINAHL 966 retrieved	S1-AB violence or AB aggression (results = 17640)
	S2-AB treating or AB intervention or AB managing or AB preventing or AB prevention or AB management or AB de-escalation or AB reducing (results = 424521)
	S3-AB hospital or AB clinic or AB ward or AB unit or AB department (results = 286193)
	S4-S1 AND S2 AND S3. Limiters-English language; age groups: all adult; language: English (results = 514)

PsycINFO 1512 retrieved	S1-AB violence OR AB aggression (results = 100210)
	S2-AB treating OR AB intervention OR AB managing OR AB management OR AB prevention OR AB preventing OR AB de-escalation OR AB reducing (results = 613675)
	S3-AB hospital OR AB clinic OR AB ward OR AB unit OR AB department (results = 263109)
	S4-S1 AND S2 AND S3. Narrow by language: English. Narrow by subject age: adulthood (18 years and older) (results = 1285)

Cochrane 45 retrieved	((“Violence”) (word variations have been searched))

**Table 3:** Table of included studies, *n* = 12.

Author (country)	Aim	Study design	Participants	Intervention	Findings
Adams et al. [8] (Australia)	To assess the effectiveness of clinical education in identifying patients at high risk of violence and reducing the frequency of such incidents	Before and after study design	Registered nurses (41 pre, 45 post), enrolled nurses (15 pre, 17 post), and assistants in nursing (3 pre, 5 post). A total of 65 pre and 73 post	An education program addressed four key areas (assessment, planning, implementation (crisis), postincident). Case studies and inpatient scenarios provided context, immediacy, and relevance, and 77% of the staff completed the program	Posteducation, knowledge increased significantly (*p* = 0.001, CI 0.256–0.542), the use of verbal deescalation increased significantly (*p* = 0.011, 1df) and the frequency and recurrence of incidents decreased. All perpetrators met criteria indicating a high risk for violence
Arnetz et al. [15] (United States)	To evaluate the effects of a randomised controlled intervention on the incidence of patient-to-worker (type II) violence-related injury in hospitals	Randomised controlled trial	15000 employees	41 units across 7 hospitals were randomised into intervention (*n* = 21) and control (*n* = 20) groups. Intervention units received unit-level violence data to facilitate development of an action plan for violence prevention; no data were presented to control units	Six months postintervention, incident rate ratios of violent events were significantly lower on intervention units compared with controls (incident rate ratio (IRR) 0.48, 95% confidence interval (CI) 0.29 to 0.80). At 24 months, the risk for violence-related injury was lower on intervention units, compared with controls (IRR 0.37, 95% CI 0.17 to 0.83)
Baig et al. [29] (Pakistan)	To assess the effectiveness of training in prevention, deescalation and management of verbal and physical violence in healthcare settings by equipping healthcare providers with essential skills	A quasiexperimental study using mixed method concurrent embedded design	154 healthcare providers working in emergency, gynaecology, and obstetrics departments	4-hour deescalation training	The overall self-perceived mean score of confidence in coping with patient aggression instrument “(CCPAI)” scale was significantly higher in intervention group (mean = 27.49, SD = 3.53) as compared to control group (mean = 23.92, SD = 4.52) (*p* < 0.001). No statistically significant difference was observed between intervention and control groups with regard to the frequency of violence faced by HCPs posttraining and major perpetrators of violence
Drummond et al. [27] (United States)	To describe one general hospital's success in reducing violent behaviour amongst repetitively disruptive patients	Comparison study	48 patients	An instrument was designed by the medical centre's multidisciplinary behavioural emergency committee (BEC) so employees could report incidents involving an act or threat of violence that disrupted patient care	The number of incidents declined by 91.6%, and visits to the medical centre for any reason decreased by 42.2%. The ratio of violent incidents to visits after the program was begun was less than one-sixth the rate before the program
Ford [11] (United States)	To determine if one hour of deescalation and self-defence training can reduce violence and improve the work environment for patient care providers; thereby allowing improved patient care quality	Literature review and intervention evaluation	Representatives from two nursing units, human resources, risk management, security, and administration^*∗*^	Research was initiated by developing an intervention utilising violence theory and constructs	In conclusion, the results are mixed and statistically inconclusive. From the care providers' perspective, any reduction in violence is significant. The data regarding the training interventions indicate that there was an empirical, albeit not statistically significant, change in Code Gray reports. Training may have reduced the violence in the eldercare unit by nearly half
Gillam [10] (United States)	To evaluate the nonviolent crisis intervention training investment	Qualitative quality improvement observational study	75,246 emergency department visits with 111 ED code purple events from November 2012 to October 2013	Nonviolent crisis intervention (NCI) training was initiated to reduce the incidence of violence in an acute care hospital ED with more than 75000 annual visitors	There was a negative correlation between violence and NCI training in the previous 90–150 days; regression determined a 23% decrease in code purples, pursuant to training
Jouybari et al. [31] (Iran)	To determine the effect of anger management training on controlling the perceived violence and aggression of nurses in emergency departments	The quasiexperimental study, pretest-posttest study design, and two groups	112 nurses in emergency departments of educational healthcare centres of Gorgan, Iran, in 2017	Nurses were randomly divided into test and control groups. In the test group, training in anger management skills was carried out in person, followed by 2-month virtual training, including short messages related to the skill of anger management delivered via telegram. No specific measure was obtained in the control group. The subjects filled out an anger management skill questionnaire before and after the intervention	The comparison of exposure level to verbal violence in the control group was low at the beginning of the study, which had a significant increase at the end of the investigation (*p* = 0.001). There was a significant difference between the level of exposure to physical violence in the test group before and after the intervention (*p* = 0.007), whereas no change was observed in the control group (*p* = 0.91). Only in the test group, there was a reduction in the level of exposure to sexual anger, and the difference was statistically significant (*p* = 0.006)
Kling et al. [9] (United States)	To examine the use and effectiveness of the alert assessment form	Chart review and assessment	117 violent patients' charts were reviewed and compared to 161 nonviolent patient charts	The alert assessment form is part of the alert system, used by one large acute care hospital to identify patients with a propensity for violence	The overall use of the alert assessment form for violent and nonviolent patients was 75.7% and 35.4%, respectively. The assessment form was found to have moderate sensitivity (71%) and high specificity (94%). It is reasonably effective in identifying potentially violent or aggressive patients when it is used according to protocol
McDade [32] (United States)	To examine the efficacy of an aggression management training in managing aggression and violent behaviour at East Mississippi State Hospital (EMSH), an inpatient behavioural health program	Investigative study	3616 presentations. Hospital staff	The efficacy of the Mandt system was examined through 4 key variables: patient-to-patient incidents, patient-to-staff incidents, seclusion episodes and restraint episodes. Over a 6-year period, incidents of aggression were identified by extracting archival data from incident reports. Archival data were examined 3 years prior to the implementation of the Mandt system and 3 years after the implementation of the training	The researcher found that the rate of patient-to-patient incidents decreased as well as the rate of seclusions and restraint episodes following the implementation of the Mandt system training. The rate of the patient-to-staff incidents did not decrease. Effective training on the management of aggression is essential in decreasing aggressive and violent behaviour
Peek-Asa et al. [33] (United States)	Compares surveys of emergency nurses before and after the implementation of the AB508	Cross-sectional survey	17 California emergency departments	The California Emergency Nurses Association (ENA) Government affairs committee conducted a survey of California emergency departments in 1990 with a follow-up survey in 2000	Most hospitals reported fewer violent episodes after the implementation of AB508. However, 32% of hospitals reported that 5 or more verbal threats occurred monthly, and 5% reported that 5 or more violent injuries occurred monthly. Overall, hospitals reported improvements in security programs. The most notable increase was in employee training, which rose from 34% to 95.6% of reporting hospitals. However, almost a quarter of hospitals reported not having general violence prevention policies
Sharifi et al. [34] (Iran)	To evaluate the effects of an education program, risk assessment checklist, and preventative protocol on violence against emergency department nurses	Quasiexperimental intervention before and after the study	37 nurses in the emergency department of Tohid Hospital	A workshop in which emergency department nurses were taught a method of using a risk assessment checklist and preventative protocol. The intervention lasted six weeks	The mean score of violence before the intervention was 8.4, and after the intervention, it was 2.7, which was statistically a significant difference (*p* < 0.0001). In addition, there were significant differences in the mean frequency of verbal abuse (*p* < 0.0001), assessment of workplace security (*p* = 0.006), fear of injury (*p* < 0.02) and type of reaction to violence (*p* < 0.01) before and after the intervention amongst the nurses
Touzet et al. [35] (France)	To assess the impact of a comprehensive prevention programme aimed at preventing incivility and verbal violence against healthcare professionals working in the ophthalmology ED of a university hospital	Single-centre, prospective interrupted time-series study	Seven nurses, six ward aides, two orthoptic students, seven residents in ophthalmology, and four senior ophthalmologists	3 periods; a three-month preinterventional period, a three-month training period and a twelve month implementation period of the prevention programme	There were a total of 22 107 admissions, including 272 (1.4%) with at least one act of violence reported by the healthcare workers. Almost all acts of violence were incivility or verbal harassment. The rate of violence significantly decreased from the preintervention to the intervention period (24.8, 95% CI 20.0 to 29.5, to 9.5, 95% CI 8.0 to 10.9, acts per 1000 admissions, *p* < 0.001). An immediate 53% decrease in the violence rate (incidence rate ratio = 0.47, 95% CI 0.27 to 0.82, *p* = 0.0121) was observed in the first month of the intervention period, after the implementation of the triage algorithm

^*∗*^no *n* = provided.

**Table 4:** Quality Assessment for Diverse Studies (QuADS).

Study	Theoretical or conceptual underpinning to the research	Statement of research aims	A clear description of the research setting and target population	The study design is appropriate to address the stated research aim/s	Appropriate sampling to address the research aim/s	Rationale for choice of data collection tool/s	The format and content of the data collection tool are appropriate to address the stated research aim/s	Description of the data collection procedure	Recruitment data provided	Justification for the analytic method selected	The method of analysis was appropriate to answer the research aim/s	Evidence that the research stakeholders have been considered in the research design or conduct	Strengths and limitations critically discussed
	Score	Score	Score	Score	Score	Score	Score	Score	Score	Score	Score	Score	Score
Adams et al. [8]	0	3	3	2	3	2	2	2	2	2	3	0	1
Arnetz et al. [15]	0	3	3	3	3	2	2	2	2	2	3	0	1
Baig et al. [29]	0	3	3	2	3	2	2	2	2	2	3	0	1
Drummond et al. [27]	0	3	3	2	3	0	3	2	2	2	3	0	0
Ford [11]	0	3	3	2	3	1	2	2	2	2	3	0	1
Gillam [10]	0	3	3	2	3	1	2	2	2	2	3	0	1
Jouybari et al. [31]	0	3	3	2	3	2	2	3	2	2	3	0	1
Kling et al. [9]	0	3	3	2	3	2	2	2	2	2	3	0	1
McDade [32]	0	3	3	2	3	2	2	2	2	2	3	0	1
Peek-Asa et al. [33]	0	3	3	2	3	2	3	2	2	2	3	0	1
Sharifi et al. [34]	0	3	3	2	3	2	2	3	2	2	3	0	1
Touzet et al. [35]	0	3	3	2	3	2	2	3	2	2	3	0	1

0 = not at all, 1 = very slightly, 2 = moderately, 3 = complete.

## Data Availability

All data including protocols are included within the article.

## Additional Points

This study is registered with PROSPERO.
